# Exploring Various Techniques for the Chemical and Biological Synthesis of Polymeric Nanoparticles

**DOI:** 10.3390/nano12030576

**Published:** 2022-02-08

**Authors:** Thiruchelvi Pulingam, Parisa Foroozandeh, Jo-Ann Chuah, Kumar Sudesh

**Affiliations:** Ecobiomaterial Research Laboratory, School of Biological Sciences, Universiti Sains Malaysia, Gelugor 11800, Penang, Malaysia; thiruchelvi@usm.my (T.P.); parisa.forooz@gmail.com (P.F.); jannchuah@gmail.com (J.-A.C.)

**Keywords:** polymeric nanoparticles, nanoprecipitation, emulsification solvent evaporation, emulsification solvent diffusion, polyhydroxyalkanoates (PHA), natural nanoparticles

## Abstract

Nanoparticles (NPs) have remarkable properties for delivering therapeutic drugs to the body’s targeted cells. NPs have shown to be significantly more efficient as drug delivery carriers than micron-sized particles, which are quickly eliminated by the immune system. Biopolymer-based polymeric nanoparticles (PNPs) are colloidal systems composed of either natural or synthetic polymers and can be synthesized by the direct polymerization of monomers (e.g., emulsion polymerization, surfactant-free emulsion polymerization, mini-emulsion polymerization, micro-emulsion polymerization, and microbial polymerization) or by the dispersion of preformed polymers (e.g., nanoprecipitation, emulsification solvent evaporation, emulsification solvent diffusion, and salting-out). The desired characteristics of NPs and their target applications are determining factors in the choice of method used for their production. This review article aims to shed light on the different methods employed for the production of PNPs and to discuss the effect of experimental parameters on the physicochemical properties of PNPs. Thus, this review highlights specific properties of PNPs that can be tailored to be employed as drug carriers, especially in hospitals for point-of-care diagnostics for targeted therapies.

## 1. Introduction

Nanoparticles (NPs) are defined as particles with all three dimensions confined within the range of 1 to 100 nm [1,2,3,4]. The growing attention towards NPs stems from the fact that their mechanical, chemical, optical, electrical, and magnetic properties differ from those of their bulk counterparts, and these properties can be altered by varying the size of NPs [5,6]. NPs are of great interest in a variety of sectors, including physics, agriculture, chemistry, engineering, electronics, biology, food technology, medicine, and bioengineering, due to their small size and ability to tailor their properties for specific requirements [7,8,9,10,11,12,13,14,15,16,17].

NPs offer the perfect characteristics for delivering therapeutic medications to the body’s target sites [18]. In contrast to micron-sized particles that are rapidly eliminated by the immune system, NPs demonstrated much higher efficiency as drug delivery carriers [19,20,21]. Because of their larger surface area, NPs can effectively penetrate cells and traverse the blood–brain barrier and they are easily destroyed [22,23,24]. NPs can be produced using a variety of natural and synthetic materials, which are biodegradable or non-biodegradable [25]. Examples of NPs include solid–lipid nanoparticles, silver nanoparticles, gold nanoparticles, magnetic nanoparticles, mesoporous silica nanoparticles, nanocrystals, carbon nanotubes, albumin nanoparticles, fullerene nanoparticles, and polymeric nanoparticles (PNPs).

Many types of NPs have been investigated for clinical use but have not been accepted widely due to their toxicity to some extent [26]. Biopolymers are employed in the manufacturing of NPs for biomedical applications to avoid cytotoxicity concerns [27,28]. Biopolymers are well-known for being non-toxic, biodegradable, and biocompatible [29,30]. Depending on the intended uses, PNPs can be simply and cost-effectively generated on a wide scale using a variety of technologies. PNPs have applications in different fields such as electronics [31], photonics [32], environmental technology [33], medicine [34], bio-imaging [35], diagnostics [36], biotechnology [37], biomedical drug delivery [38,39,40], and energy harvesting [41].

Due to their subcellular size, biodegradability, biocompatibility with tissue and cells, and controlled and sustained-release capabilities, PNPs are attractive candidates for the delivery of vaccinations, antibiotics, and cancer treatments [42,43,44,45,46]. PNPs can enhance the bioavailability, solubility, and retention time of drugs. Moreover, PNPs do not cause any toxic, inflammatory, or immunogenic side effects [47,48]. Different polymers such as polyhydroxyalkanoate (PHA) [49,50,51,52], polylactic acid (PLA) [53,54,55], poly(lactic-*co*-glycolic acid) (PLGA) [56,57], polycaprolactone (PCL) [58,59,60], polyglycolide (PGA) [61], polyanhydride [62], polycyanoacrylate [63], poly glutamic acid [64], polymalic acid [65,66], poly(N-vinyl pyrrolidone) [66,67], poly(methyl methacrylate) (PMMA) [68,69], poly(vinyl alcohol) [70,71], poly(acrylic acid) [72,73], poly acrylamide [74,75], and poly(methacrylic acid) [76,77] have been used for the synthesis of PNPs.

This review paper describes the different methods used for producing PNPs and how variation in experimental parameters can enable the control of NP properties. As PNPs are colloidal systems made up of natural or synthetic polymers, their synthesis methods are generally categorized into two groups. They are (1) the direct polymerization of monomers (emulsion polymerization, surfactant-free emulsion polymerization, mini-emulsion polymerization, micro-emulsion polymerization, and microbial polymerization) and (2) the dispersion of preformed polymers (e.g., nanoprecipitation, emulsification solvent evaporation, emulsification solvent diffusion, and salting-out). Table 1 describes the advantages and limitations of these two types of polymer synthesis methods.

## 2. Methods for Producing PNPs

PNP preparation can be divided into two categories: monomer polymerization and preformed polymer dispersion [96,97,98]. Emulsion polymerization, surfactant-free emulsion polymerization, mini-emulsion polymerization, and micro-emulsion polymerization are all processes that can be used to polymerize monomers [99,100]. Likewise, nanoprecipitation, emulsification solvent evaporation, emulsification solvent diffusion, and salting-out can all be utilized to make PNPs from preformed polymers [101,102,103]. The type of polymer, size requirement, and application region all influence the method of preparation [104,105]. The technique of preparation is crucial to achieving the desired qualities. PNPs made for biological applications, for example, should be free of additives and reactants [106].

The type of polymer used determines the features of the produced NPs that are designed for a certain purpose [107,108]. The drug delivery capabilities of PLGA and poly(3-hydroxybutyrate) P(3HB) were studied by employing docetaxel (DTXL). Although the toxicity profiles of P(3HB) and PLGA were similar, P(3HB) had a nearly two-fold higher loading efficacy and poorer retention rates than PLGA [109]. Dissolution, solubility, cellular uptake, release of drugs, bio-distribution, and circulatory half-life are all influenced by the size of PNPs [110,111,112]. The challenge in the preparation of PNPs is the ability to produce uniform particles to have consistent performance [113,114]. NPs with a broad size distribution result in difficulty in establishing their applications [115].

### 2.1. Formation of NPs from Preformed Polymers

This section discusses the many ways to make PNPs from pre-formed polymers, including nanoprecipitation, emulsification solvent evaporation, emulsification solvent diffusion, and salting-out [101,102,103]. The initial stage in all of these approaches is to prepare an emulsification system, which is the same for all of them. The second step is the formation of PNP, which is different for each method. The name of the method is conferred by the principles of the second step, which can occur either by precipitation or by the evaporation of the organic solvent [116,117].

#### 2.1.1. Nanoprecipitation

Fessi et al. devised the nanoprecipitation approach, often known as the solvent displacement, antisolvent precipitation, solvent shifting, and desolvation methods, for the creation of PNPs in 1989 [118]. Nanoprecipitation is a simple, easy, fast, and reproducible single-step method. This approach does not demand a lot of energy and can be scaled up simply [119]. Nanoprecipitation is time-efficient, inexpensive, and does not need a precursor emulsion like other methods [120]. The size of the NPs generated by this approach is changed by altering the parameters, and they are small with a limited size distribution [121]. Nanoprecipitation is based on interfacial deposition, in which the transport of a solvent into a non-solvent causes the polymer to dissolve, leading to nuclei growth, crystal growth, and nanoprecipitation [122,123,124].

**Figure 1:**
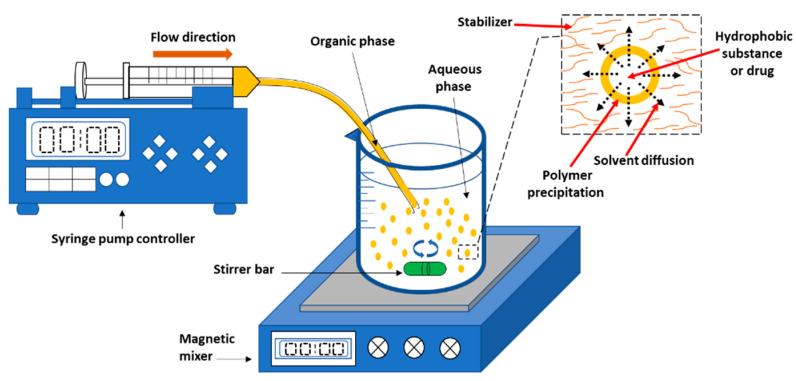
The nanoprecipitation process illustrated in a diagram. The enlarged image (inset) illustrates the process of nanoparticle (yellow spheres) formation owing to the surface tension difference between the aqueous phase (high surface tension) and organic phase (low surface tension). Adapted from Wang et al. (2016) [78].

An organic phase is introduced to the aqueous phase during nanoprecipitation. The polymer and water-miscible organic solvent, which must be miscible in the aqueous medium, make up the organic phase, which has a diffusion effect [125,126,127,128,129,130,131]. To slow aggregation, the polymer must be insoluble in the aqueous solution, which might contain a stabilizer like a surfactant [132,133,134,135]. Dropwise addition of the organic phase to the aqueous phase with moderate agitation produces NPs [136,137]. Ultracentrifugation is used to collect the NPs, which are subsequently rinsed with water to remove the surfactant. The organic solvent evaporates, hardening the NPs, which are subsequently recovered by filtering, spinning, or freeze-drying [138,139]. Organic solvents that evaporate easily such as ethanol, acetone, hexane, or methylene chloride should be chosen as a polymer solvent. Binary solvent blends such as combinations of acetone with either ethanol or methanol can also be used. Likewise, a mixture of non-solvents can be used to form NPs in this method [140,141,142]. Figure 1 shows a schematic illustration of the nanoprecipitation process. According to Quintanar et al., the difference in surface tensions induces intrafacial turbulence and thermal disparities in the system, resulting in the production of continuous solvent eddies at the interface of both liquids. When the polymer aggregates on the hydrophobic drug surface as the solvent runs away (solvent diffusion) from low surface tension regions, nanocapsules are generated. [97].

The polymer content, variety of solvent and non-solvent, proportion of solvent to non-solvent, rate of the addition of solvent to non-solvent, the effect of the stabilizer, and stirring speed all influence the size of NPs [143,144]. Due to the increase in viscosity that hinders polymer diffusion from the solvent to the non-solvent, an increase in polymer concentration leads to the creation of bigger nanoparticles [144]. Smaller NPs in a narrow size range are produced by solvents with high diffusion coefficients, such as acetone and acetonitrile [145]. It has also been established that a decrease in the solvent-to-non-solvent-volume ratio results in smaller NP sizes [146]. The nature of the stabilizer and its concentration has been shown to influence the size of NPs [147,148].

A study found that increasing the amount of surfactant (e.g., Pluronic) reduced the size of NPs by lowering interfacial tension [149]. In addition to that, employing a surfactant in the nanoprecipitation method is not necessary, enabling the production of surfactant-free particles [150]. Meanwhile, higher stirring rates have been found to produce smaller NPs [151,152]. Zhang and colleagues demonstrated that raising the stirring speed from 300 to 1200 rpm reduces particle diameter from 800 to 300 nm [153]. Specifically, low external energy input is sufficient for the nanoprecipitation method, hence a moderate stirring speed is required instead of a high stirring speed that raises the temperature [154,155,156].

NP formation using the nanoprecipitation method occurs through three different steps; particle nucleation, molecular growth and particle agglomeration [157]. Nucleation takes place when the polymer concentration reaches the saturation level, i.e., when the polymer solute in the solution is more than the amount that the solvent can dissolve [158]. The mean particle size increased significantly as the polymer concentration was increased [159,160]. Molecular growth and particle agglomeration occur with a release of energy [161,162].

Chorny and coworkers used the nanoprecipitation approach to make PLA NPs loaded with tyrphostin. Particle size increases from 70 nm to 140 nm when the polymer concentration is increased from 100 mg (5 mg/mL) to 300 mg (15 mg/mL) [163]. NPs of poly(lactide)-poly(ethylene glycol)-poly(lactide) (PLA-PEG-PLA) were synthesized by the nanoprecipitation method under different conditions. It was discovered that increasing the agitation rate resulted in a reduction in particle size [164]. Meanwhile, in another study comparing two methods for NP preparation, the nanoprecipitation method was found to be more efficient for preparing PLGA NPs encapsulating cucurbitacin compared to using the emulsion solvent evaporation method [165].

In the preparation of cellulose NPs loaded with mefenamic acid [166] and PLGA NPs loaded with N-acetylcysteine (NAC), the solvent/nonsolvent ratio, the concentration of polymer and the choice of solvent as well as nonsolvent were found to affect the size of NPs. The nanoprecipitation method, in addition to efficiently entrapping hydrophobic molecules, also has a great potential as an alternate entrapment method for hydrophilic chemicals, according to the findings [167]. Chidambaram et al. proposed changes to the traditional nanoprecipitation process in order to reduce NP size and create NPs with a narrow size distribution. They used sonication to prepare both the organic and aqueous phases, yielding Eudragit E100 NPs with a particles size of 114 nm and a uniformity of 0.259 [168].

Three distinct proteins (tetanus toxoid, lysozyme, and insulin) were entrapped in poly(d,l-lactic acid) and poly(d,l-lactic-co-glycolic acid) NPs using modified nanoprecipitation and double emulsion (w1/o/w2) techniques in a separate investigation. The use of miscible organic solvents like dimethylsulfoxide (DMSO) rather than conventional organic solvents like acetone or ethanol, as well as non-solvents like methanol or ethanol rather than water, have all been added to the nanoprecipitation process. Nanoprecipitation proved to be a suitable option to the extensively employed double emulsion approach. Nanoprecipitation was found to be the best approach for protein trapping in small, densely loaded NPs [169]. Luo et al. applied a combination of electrospraying and nanoprecipitation to produce multifunctional superhydrophobic polymethylsilsesquioxane (PMSQ) NPs with sizes smaller than 100 nm [170].

Additionally, continuous flow microfluidics is a great solution for nanoprecipitation operations, enhancing product controllability, homogeneity, and reproducibility. Nanoprecipitation through a hydrodynamic flow-focusing microchannel was used to synthesize PLGA-poly(ethylene glycol) nanoparticles (PLGA-PEG NPs). Variations in flow rates, polymer concentration, and polymer composition can be used to obtain the preferred size, drug loading, and polydispersity of the synthesized product [171]. Polycaprolactone (PCL) nanoparticles, which are biodegradable and have a tremendous potential for controlled drug delivery, were synthesized through a similar nanoprecipitation process [172]. Moreover, this technique may be used to assemble other polymers like chitosan, heparin, and hyaluronic acid in microfluidic devices, especially to produce PNPs for controlled release as well as drug delivery [173].

**Figure 2:**
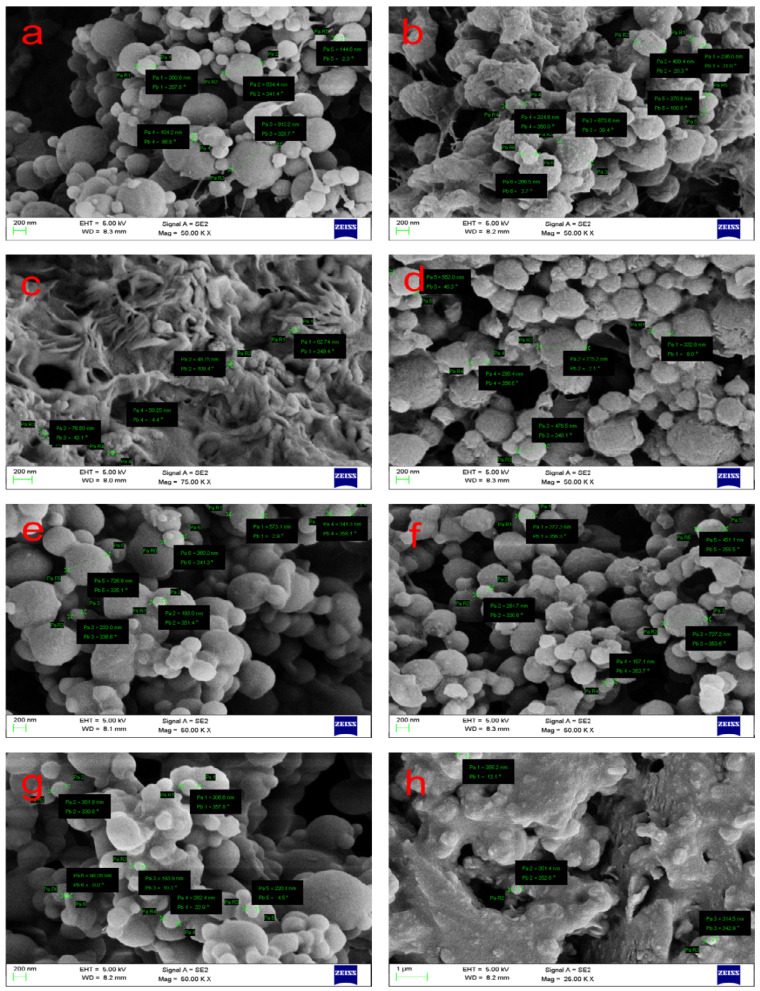
Scanning electron micrographs of synthesized P(3HB) NPs. (**a**–**d**) NPs were prepared using chloroform and (**a**) DMSO, (**b**) DMSO (loaded), (**c**) water (**d**) water (loaded), (**e**–**h**) ethyl acetate, and (**e**) DMSO, (**f**) DMSO (loaded) (**g**) water, (**h**) water (loaded). Adapted from Senthilkumar et al. (2018) [174].

Meanwhile, P(3HB) NPs were prepared by nanoprecipitation with a variety of solvent/non-solvent combinations such as ethyl acetate:DMSO, chloroform:water, chloroform:DMSO, and ethyl acetate:water. In the reported study, spherically shaped P(3HB) NPs with sizes ranging from 40 to 100 nm were successfully formed while the size of loaded PNPs were typically between 200 to 600 nm as shown in Figure 2 [174]. In another attempt, P(3HB) NPs were prepared by nanoprecipitation with a low concentration of Tween 80 as a surfactant. The size and size distribution of NPs decreased as the amount of Tween 80 in water increased to 1% (*v*/*v*) [175].

In all the examples mentioned above, P(3HB) is initially biosynthesized by microorganisms and stored in the microbial cell cytoplasm. The produced and accumulated natural polyester is then removed from the bacterial cells using suitable solvents like chloroform and purified by reprecipitation in a non-solvent like methanol. The purified P(3HB) can be mixed in solvents and used in the nanoprecipitation process to make NPs.

#### 2.1.2. Emulsification-Solvent Evaporation

The first and most extensively used method for the synthesis of PNPs is emulsification-solvent evaporation. The first step involves emulsifying the polymer solution into an aqueous phase, and the second step entails the evaporation of the solvent, which results in polymer precipitation, resulting in the production of NP [176,177,178]. The first step is to form the emulsions, which can occur by either of two main strategies. The first strategy is to produce single-emulsions, i.e., oil-in-water (o/w) and the second one is to produce double-emulsions, i.e., water-oil-water (w/o/w) or oil-water-oil (o/w/o) [179,180]. In a double emulsion, the primary emulsion (w_1_/o) is first prepared by dispersing the aqueous phase in an immiscible organic solvent containing the polymer. Subsequently, the primary emulsion is homogenized in an outer aqueous phase containing the emulsifier using a high-shear homogenizer to form the organic phase and then emulsified in the aqueous phase containing a surfactant [181,182,183,184,185,186].

**Figure 3:**
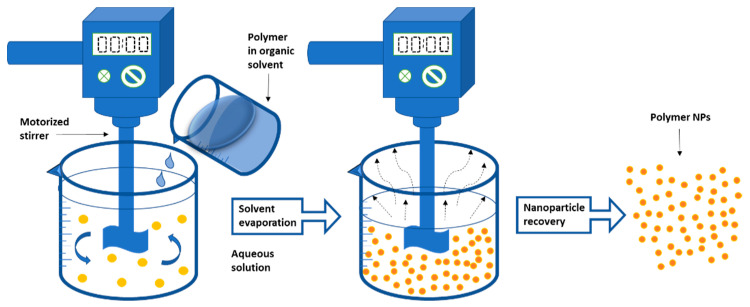
The emulsification-solvent evaporation technique is depicted schematically. Adapted from Wang et al. (2016) [78].

The solvent is then continuously evaporated while the NPs are recovered by ultracentrifugation [187,188]. The NPs are thoroughly rinsed with water and then lyophilized to remove the surfactants [189,190,191]. Figure 3 depicts a schematic illustration of the solvent evaporation procedure. The diameter of NPs can be controlled by adjusting the stirring speed, the viscosity of the aqueous and organic phases, and the type and concentration of the dispersing agent [192]. The solvent evaporation approach was used by Musyanovych et al. to make poly(l-lactide) (PLLA), PLGA, and poly(caprolactone) (PCL) NPs. The size of the NPs produced is affected by the type of polymer used. The smallest particle size was found in PLGA NPs, whereas the highest particle size was found in PCL NPs [192].

Another study generated haloperidol-loaded PLGA/PLA NPs and found that raising the polymer concentration from 5 to 66.6 mg/mL improved the NP size from 200 to 300 nm while retaining a unimodal particle-size distribution. It was discovered that lowering the solvent/non-solvent volume ratio reduced the size of PLGA/PLA NPs [193]. Bilati et al. examined at how the sonication procedure affected the properties of poly(lactide-*co*-glycolide) nanocapsules made by the water-in-oil/water solvent evaporation method. The second mixing step’s sonication time (for w/o/w emulsion) has a bigger impact on the final NP size than the first step’s sonication duration (for water-in-oil emulsion). [194].

Poly(d,l-lactide-*co*-glycolide) NPs containing praziquantel were produced by employing methylene chloride or ethyl acetate, separately, as an organic solvent in the dispersion phase. The size of methylene chloride-prepared NPs was larger than that of ethyl acetate-prepared NPs [195]. When ethanol was used as a solvent and Pluronic F-108 was used as a stabilizer, the average size of poly(ethylene oxide) (PEO) NPs generated using the single-emulsion approach was 100 to 150 nm. It was evident that the polymer concentration influenced the characteristics of the PEO NPs [196].

By modifying experimental conditions such as homogenization rate, surfactant concentration, and polymer/solvent ratio, poly(3-hydroxybutyrate-*co*-3-hydroxyhexanoate) [P(3HB-*co*-3HHx)] NPs could be produced in the size range of 180 nm to 1.5 µm. The size of P(3HB-*co*-3HHx) NPs decreased as the surfactant concentration and homogenization rate increased, whereas P(3HB-*co*-3HHx) NP size increased by increasing the polymer to solvent ratio [197]. When the ultrasound exposure period, amplitude, and exterior aqueous phase volume were increased, PCL NPs generated using the double emulsion solvent evaporation method showed a decrease in particle size. The size of NPs grew from 235 to 748 nm when the concentration of PCL was raised from 1 to 5 g. Meanwhile, the size of PCL NPs decreased with increasing surfactant (e.g., PVA) concentration from 0.05 to 0.2% [198].

Folate-targeted poly(3-hydroxybutyrate-*co*-3-hydroxyoctanoate) P(3HB-*co*-3HO) NPs were prepared by the w_1_/o/w_2_ solvent evaporation method. These NPs were loaded with doxorubicin (DOX), a chemotherapeutic drug in cancer treatment. An in vivo antitumor study of the NPs revealed a great potential of these NPs to improve the sustained release profile of doxorubicin [199]. This approach produced PEG end-capped P(3HB-*co*-3HHx) with a particle size of roughly 200 nm, which showed promise as a nanocarrier for sustained rapamycin delivery with increased cellular absorption and kinase inhibitory efficacy [200]. In addition to the P(3HB-*co*-3HO) NPs, poly(3-hydroxyvalerate-*co*-4-hydroxybutyrate) P(3HV-*co*-4HB) NPs could also be synthesized by the emulsification–solvent evaporation method. It was found that the cisplatin-loaded NPs accumulated more efficiently in the tumor cells and had a higher tumor regression effect than freely administered cisplatin, indicating that this nanocarrier was suitable for drug delivery applications [201]. Curcumin was loaded into the P(3HB-*co*-3HHx) NPs for use in breast cancer treatment. Higher drug release and better decline in tumor cell activity were observed in curcumin-loaded P(3HB-*co*-3HHx) NPs than curcumin alone, indicating that the P(3HB-*co*-3HHx) NPs are a promising tool to enable the sustained and controlled release of some drugs [202].

As a nanocarrier for ellipticine, poly(3-hydroxybutyrate-*co*-3-hydroxyvalerate) P(3HB-*co*-3HV) NPs were produced by solvent evaporation (EPT). In an in vitro test, the percentage of inhibition for EPT-PHBV NPs was around two times that of free EPT, showing that P(3HB-*co*-3HV) NPs are a viable vehicle for the administration of hydrophobic medicines for cancer treatment [203]. For cisplatin delivery, poly(4-hydroxybutyrate)-mPEG (P(4HB)-mPEG nanocarriers were developed. The cisplatin-loaded P(4HB)-mPEG NPs were shown to be more effective than free cisplatin, demonstrating that the P(4HB)-mPEG) nanocarriers are effective in delivering cisplatin to cancer cells [204].

#### 2.1.3. Emulsification Solvent Diffusion

**Figure 4:**
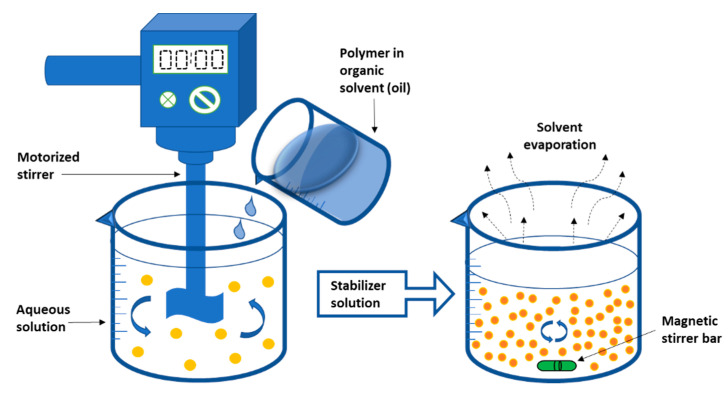
Diagrammatic representation of the emulsification solvent diffusion method.

Leroux et al. were the first to propose the emulsification-solvent diffusion approach. To start, the polymer is dissolved in an organic solvent that is saturated with water, generating an organic phase. The organic phase is then emulsified in the aqueous solution, resulting in solvent diffusion and NP production [78,135]. To precipitate the polymer, it is necessary to dilute the solvent with extra water to improve its diffusion. Lastly, the solvent is eliminated by distillation or crossflow filtration [205,206,207,208]. The aqueous phase contains a stabilizer, and the dilution phase is often water. This process has the benefit of not necessitating a homogenizer, having excellent reproducibility, and being simple to scale up [209,210]. The drawback of this procedure is that it requires a large amount of water to be eliminated from the suspension [211]. Figure 4 shows a schematic illustration of the emulsification solvent diffusion process.

Quintanar et al. proposed a mechanism for NP formation in which each droplet forms several NPs [209]. Perez et al. and Ma et al. then proceeded to modify the method suggested by Quintanar et al. for the nanoencapsulation of hydrophilic active substances. In their proposed method, the aqueous inner phase includes an active substance as well as a stabilizing agent such as PVA or poly(vinylpyrrolidone) (PVP), while the external phase comprises the polymer and organic solvent. The emulsion was initially diluted with the solvent (ethanol), resulting in organic solvent migration. Then, water was added to facilitate the collection of NPs [212,213]. Hassou and Moinard-Chécot et al. used a step-by-step diffusion analysis using the stopped-flow methodology to represent different states that occur in the emulsification solvent diffusion method during the dilution stage. It was discovered that the solvent diffuses quickly from the droplets, taking less than 20 ms [214,215]. Pramual et al. formulated 5,10,15,20-Tetrakis(4-hydroxy-phenyl)-21H, 23H-porphine pTHPP (hydrophobic photosensitizer) loaded P(3HB-*co*-3HV) NPs for photodynamic therapy (PDT) by the emulsification-diffusion method. The size distribution of P(3HB-*co*-3HV) NPs was narrow, ranging from 169.0 to 211.2 nm. The pTHPP-loaded P(3HB-*co*-3HV) NPs exhibited high photocytotoxicity towards HT-29 human colon cancer cells compared to pTHPP alone. These results indicated that the P(3HB-*co*-3HV) NPs are potential vehicles for the delivery of hydrophobic photosensitizer drugs in photodynamic therapy [216]. PHA NPs encapsulating TGX221 anti-cancer drugs were also developed. TGX221 was slowly liberated from the PHA-based NP and proliferation in NP-TGX221-treated cells was considerably slower than in cells receiving free TGX221 [217].

Using a modified emulsification solvent diffusion process, Chen et al. developed curcumin-loaded PLGA (PLGA-Cur) NPs with a mean range of 190 nm. Anti-tumor activity was successfully detected following the delivery of PLGA-Cur NPs into cells, and in comparison, with free curcumin, PLGA-Cur NPs demonstrated the increased inhibition of HL60 and HepG2 cancer cells with lower IC50 values. Moreover, confocal microscopy analysis showed that the curcumin-loaded PLGA NPs increased apoptosis in cancer cells when compared with free curcumin [218]. PCL NPs were made using ethyl acetate as the solvent and PVA as the stabilizing agent, respectively. The polymer concentration, solvent volume, type and amount of the surfactant, as well as the concentration of oil in the organic phase, were all observed to affect the size of PCL NPs [219]. The solvent and stabilizing agents utilized to make PLA NPs were ethyl acetate and Pluronic F68, respectively. Particle size increased from 260 to 530 nm as PLA content increased [220]. In another investigation, as the amount of surfactant was raised, the size of PCL NPs shrank [221]. A comparison made using different stabilizers, di-dodecyl dimethylammonium bromide (DMAB) and PVA, for the production of PLGA NPs revealed that DMAB produced smaller PLGA NPs [222]. The influence of homogenization and sonication on the size of PLGA NPs was explored by Jain et al., who discovered that sonication resulted in smaller particles with an average size of 165 nm, whereas homogenization resulted in particles with an average size of 225 nm [223].

#### 2.1.4. Salting-Out Technique

The salting-out method is a variation of the emulsification-solvent diffusion method that makes use of the salting-out effect [224,225]. The difference between the salting-out method and the emulsion diffusion method is that the former method does not require a solvent diffusion step, due to the existence of salts [226,227]. The aqueous phase consists of water, stabilizer, and salting-out agents. Electrolytes including sodium chloride, magnesium chloride, calcium chloride, and magnesium acetate, as well as non-electrolytes like sucrose, are salting-out agents [228,229,230]. The salting-out agents should be insoluble in the organic solvent. The kind of salting-out agent used has a big impact on how well drugs are encapsulated [231,232].

**Figure 5:**
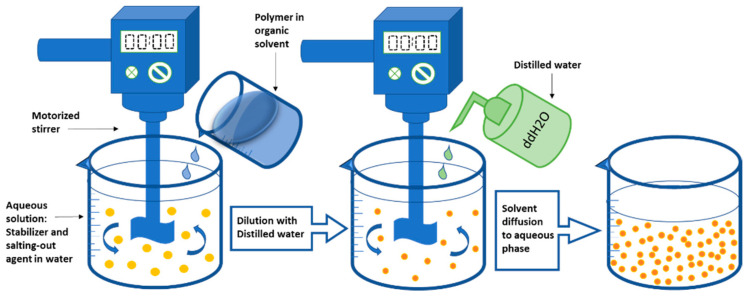
Diagrammatic interpretation of the salting-out technique.

The organic phase, which contains the polymer in a water-miscible organic solvent, is introduced to the aqueous phase in this process. The emulsion is then diluted with adequate amounts of water while constantly swirling to reduce the electrolyte’s ionic strength and improve solvent diffusion [233,234]. The generation of NPs is caused by the migration of the solvent from the organic phase to the aqueous phase during dilution. Lastly, crossflow filtering is used to remove the salting-out agent, and the produced NPs are collected [85,235,236]. The lack of chlorinated solvents, which are hazardous to the physiological system, is an advantage of the salting-out procedure. The use of salt in the preparation process necessitates purifying processes, which is a downside of this method [84,85]. A schematic representation of the salting-out technique is shown in Figure 5.

Poly(trimethylene carbonate) (PTMC) NPs were produced by single-emulsion and salting-out methods. The PTMC NPs formed by the salting-out method were smaller than those formed by the single-emulsion method. In the single-emulsion approach, the effect of polymer concentration and stirring speed on NP size was more pronounced. Another difference between these two processes is the type of organic solvents utilized; in the salting-out approach, water-miscible THF was used, whereas in the single-emulsion method, water-immiscible dichloromethane (DCM) was used [237]. Salting-out, emulsification-diffusion, and nanoprecipitation procedures were used to make methacrylic acid copolymer NPs. The size range of the methacrylic acid copolymer NPs was broader for the salting-out method (123–710 nm) compared to the emulsification-diffusion method (108–715 nm) and the nanoprecipitation method (147–245 nm) [238]. Ethylcellulose (EC) and Eudragit-S-100 (ED) NPs produced by the salting-out technique had a mean particle size and zeta potential value of 211 nm and −43.7 mV, respectively [239].

In another work, sodium chloride was used as the salting-out agent rather than magnesium chloride or magnesium acetate to make PLGA NPs. The NPs generated were spherical, measuring 111.4 ± 2.35 nm in diameter and having a modest polydispersity (0.062 ± 0.023) [240]. Meanwhile, tetrahydrofuran (THF) was used to make PLA NPs with a diameter of less than 200 nm [241], while paracetamol-loaded Eudragit S100 NPs were produced using ethanol as solvent, sodium carboxymethylcellulose as a stabilizer, and zinc sulfate heptahydrate (ZnSO_4_·7H_2_O) as the salting-out agent [242]. Zweers et al. used acetone and magnesium chloride hexahydrate (MgCl_2_·6H2O) as the organic solvent and salting-out agent, respectively, to make PEO-PLGA NPs with a size of about 200 nm [243,244]. Similarly, PLA end-capped with 1- pyrenebutanol (PLAP) NPs were synthesized using MgCl_2_·6H_2_O as the salting-out agent [245].

### 2.2. Formation of Nanoparticles by Polymerization of Monomers

The methods that were explained in the previous sections are used to produce PNPs from preformed polymers. PNPs can also be produced by the polymerization of monomers. This section explores the methods employed for the polymerization of monomers such as emulsion polymerization, surfactant-free emulsion polymerization, mini-emulsion polymerization and micro-emulsion polymerization.

#### 2.2.1. Emulsion Polymerization

**Figure 6:**
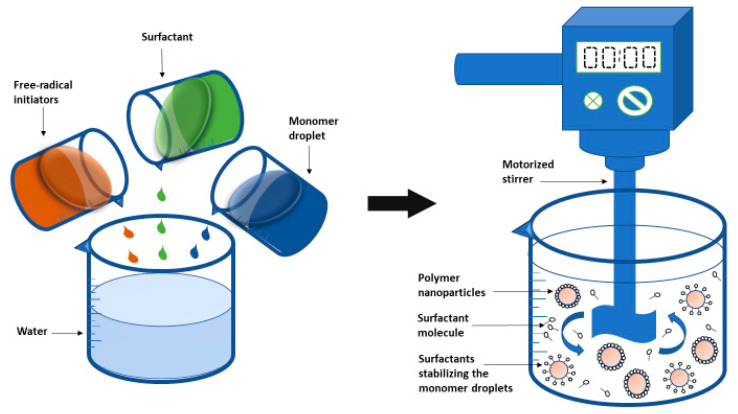
Schematic representation of the emulsion polymerization method.

Emulsion polymerization is one of the most commonly used, fastest, and readily scalable method for producing PNPs. The method of emulsion polymerization can be divided into two groups, depending on whether the continuous phase is organic or aqueous. The dispersion of monomer into an emulsion or a substance in which the monomer is not soluble is part of the continuous organic phase technique. The monomer is dissolved in a continuous phase, which is commonly an aqueous solution, without the use of surfactants or emulsifiers in the aqueous continuous phase [211,246]. Surfactant-based emulsion polymerization can be divided into two types: conventional and surfactant-free emulsion polymerization [86,247]. A water-soluble initiator, water, a somewhat water-soluble monomer, and surfactant are utilized in the traditional approach. Water is an environmentally friendly dispersion medium that also helps to dissipate heat during polymerization [107,108,248]. When a monomer dissolves in the continuous phase, initiation takes place with an initiator molecule. The initiating agent forms monomeric radicals that interact with the monomer and initiate the reaction. The radicals propagate until they reach a critical chain length, at which point their aqueous solubility is reduced. At this stage most of the surfactants are engaged in stabilization process and have formed polymer particles. However, the polymerization reaction continues until no new particles are nucleated. The termination process occurs at the end stage when there is a decrease in the polymerization rate [249,250,251]. Figure 6 depicts a schematic illustration of the emulsion polymerization technique.

Other PNPs successfully produced by the same method include polystyrene-*b*-poly[poly(ethylene glycol) methyl ether methacrylate] (PS-*b*-P(PEGMA300), and PS-*b*-P(PEGMA1100)) PNPs [252]. Using SDS as a surfactant, Garay-Jimenez et al. synthesised polyacrylate NPs by the emulsion polymerization of acrylate compounds in a mixture of butyl acrylate and styrene. Anionic, cationic, zwitterionic, and noncharged (amphiphilic) surfactants were used to create poly(butyl acrylate-styrene) emulsions. The emulsions’ cytotoxicity and microbiological activity were compared before and after purification. The findings showed that attaching a polymerizable surface to the nanoparticle matrix has no effect on the emulsion’s cytotoxic or antibacterial effects, irrespective of whether the emulsion is purified or not and that the perfect properties are associated with using non-ionic surfactants rather than those with zwitterionic, cationic, or anionic [253].

To encapsulate magnetite particles and improve particle-size distribution, emulsion polymerization was used to create magnetic polymer matrix composite nanoparticles (MPCNPs) (PSD). Transmission electron microscopy (TEM) and vibrating sample magnetometry were used to characterize MPCNPs (VSM). The results showed that the emulsion polymerization approach was successful in encapsulating magnetite particles [254]. Under microwave radiation, styrene emulsion polymerization was carried out at 70 °C using sodium dodecyl sulphate (SDS) as a surfactant and potassium persulfate (KPS) as an initiator [255]. Another study used microwave irradiation to accomplish the emulsion polymerization of methyl methacrylate (MMA) and butyl acrylate (BuA) using potassium persulfate (K_2_S_2_O_8_) as an initiator and Disponil A3065 as an emulsifier [256]. The size of polystyrene NPs produced by the ultrasonic irradiation emulsion polymerization of styrene using polymeric carboxymethyl cellulose and alkyl poly(etheroxy) acrylate (CMCA9) as surfactant was 30 to 60 nm [257]. The size of PVK NPs is regulated by the concentration of VCz. Polyvinylcarbazole (PVK) NPs were generated via emulsion polymerization of N-vinylcarbazole (VCz) [258].

#### 2.2.2. Surfactant-Free Emulsion Polymerization

Surfactants are utilized in the traditional emulsion polymerization procedure and should be eliminated from the finished product. Surfactant removal is a time-consuming operation that raises manufacturing costs [89,259]. An emulsion polymerization process without surfactants, i.e., a surfactant-free emulsion polymerization method, was devised to alleviate this limitation [260,261]. To generate PNPs, this method offers a straightforward, green approach that does not require the inclusion and subsequent removal of stabilizing chemicals. A water-soluble initiator (KPS, potassium persulfate), monomers, and water are the reagents utilized in this process. The stabilization of PNPs is achieved using ionizable initiators or ionic co-monomers in this technique [262,263,264,265].

The surfactant-free emulsion polymerization procedure using microwave irradiation produced PMMA NPs with a narrow size distribution. When the monomer concentration was increased from 0 to 0.3 mol/L, the size of the NPs rose from 103 to 215 nm [266]. The Cu^2+^/HSO_3_^−^ redox initiation system was used to commence the surfactant-free emulsion polymerization of MMA and PMMA NPs with a negative charge in the size range of 165 to 223 nm were produced [267]. PMMA NPs in the size range of 200 to 600 nm were successfully developed using hydrophilic laponite clay to stabilize methyl methacrylate emulsions dispersed in distilled water [268], while NPs with a dimension less than 100 nm and high solid content were accomplishment of this project using KPS and acetone as initiator and co-solvent, respectively [269]. Using NaSS as a stabilizing agent and water as the reaction medium, poly-acrylate NPs with fluorine and silicon in the shell with a mean range of 172.5 nm were produced [270]. By the surfactant-free emulsion polymerization of styrene utilizing ultrasonic irradiation in the presence of potassium persulfate (KPS) as an anionic initiator and cetyl alcohol as a co-stabilizer, Faridi Majidi et al. produced polystyrene NPs in the size range of 200–250 nm [271]. Surfactant-free emulsion polymerization produced poly(hydroxyethyl methacrylate) (PHEMA) NPs with a mean size of 150 nm, a polydispersity index of 1.171, and a surface area of 17,779 m^2^/g [272]. Lee et al. used Fe^3+^ catalyzed emulsion polymerization to produce poly(styrene/thiophene) NPs with particle sizes ranging from 300 to 800 nm [273]. Polyimide NPs were synthesized in a continuous phase by heterophase polycondensation of various aromatic tetra-carboxylic acids and diamines in imidazolium-based ionic liquids (IL) [274]. Kim et al. synthesized polypyrrole NPs utilizing benzene octanol and ethyl acetate as continuous phases. Changing the water and octanol volume ratios led to fewer particles with an average size of 60 nm [275].

Colloidal NPs with a PMMA or poly(butyl methacrylate) core and a cationic polymer stabilizing shell were produced using reversible addition fragmentation chain transfer-mediated surfactant-free emulsion polymerization and had hydrodynamic diameters ranging from 32 to 96 nm. The wetting behaviour of such core-shell NPs, which can be fine-tuned depending on the internal nanostructure (soft or rigid core) and external temperature, allows for the creation of controllable functional hybrid colloidal arrays [276].

#### 2.2.3. Mini-Emulsion Polymerization

**Figure 7:**
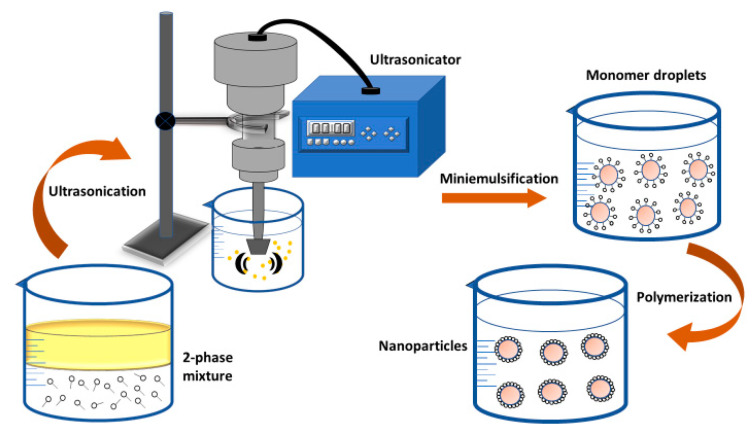
Diagram depiction of the mini-emulsion polymerization method.

The co-stabilizer, initiator, surfactant, monomer mixture, and water are all required components for the mini-emulsion process. The utilization of a low-molecular-mass co-stabilizer as well as a high-shear device such as ultrasound in this approach [91,277,278,279,280] is the fundamental distinction between mini-emulsion polymerization and emulsion polymerization. The sort of co-stabilizer and initiator used has a big impact on how the NPs develop and what they look like. Figure 7 shows a diagram illustration of the mini-emulsion polymerization process.

The mini-emulsion polymerization process was used to make a variety of PNPs. This approach produced polyacrylonitrile NPs in the size range of 100 to 180 nm using HD and SDS as the co-stabilizer and surfactant, respectively [281]. Similarly, PHEMA NPs were made with the surfactant Span 80 or KLE3729 and the co-stabilizer CH or HD. The nanoparticles produced were reported to be between 50 and 200 nm in size [282]. SDS/DMA and SDS/SMA were used, in another study, to stabilize mini-emulsion polymerizations of styrene [283]. Other examples include the composite colloidal NPs, made of magnetite as magnetic core and poly(ethyl-2-cyanoacrylate) as a polymeric shell [284]; polystyrene-single wall carbon nanotube (PS-SWNT) with SDS as surfactant and 1-pentanol as co-stabilizer [285]; and phosphonated polystyrene, as well as PMMA NPs (size range of 102 to 312 nm) produced by the free-radical copolymerization of vinylphosphonic acid (VPA) [286].

#### 2.2.4. Micro-Emulsion Polymerization

A new method for manufacturing nanosized PNPs is micro-emulsion polymerization. Despite the fact that emulsion polymerization and micro-emulsion polymerization are similar processes that form polymers with high molar mass, their kinetics differ, resulting in micro-emulsion polymerization having smaller particle sizes and fewer chains per particle [287,288,289,290]. A water-soluble initiator is introduced to the aqueous phase, which contains a lot of surfactants, in the microemulsion polymerization technique. Because initiation cannot occur simultaneously in all microdroplets, polymer chains begin to form only in some of them. Due to osmotic and elastic forces, microdroplets will collapse later, resulting in larger particles and the development of empty micelles [291,292,293,294]. In a microemulsion, polymerization kinetics, PNP properties, and the concentration and type of initiator, surfactant and monomer are determining factors [293]. Some researchers have been carried out to see how these parameters affect the characteristics of NPs.

Micro-emulsion polymerization was used to create poly(vinyl acetate) lattices with a high total solid [93]. On the micro-emulsion polymerization of vinyl acetate stabilized with Aerosol OT (AOT), the effects of temperature, concentration and type of initiator (V-50 and KPS) were investigated. It was found that the reaction rates increased with the concentration of V-50 and temperature. Furthermore, the differences in electrostatic attraction between KPS and V-50 free radicals, as well as charged micro-emulsion droplets, resulted in quicker polymerization rates for KPS [295]. A cationic surfactant (e.g., CTAB) and a non-ionic surfactant were used to make poly(dimethylsiloxane) (PDMS) NPs in the range of sizes of 12–80 nm [296]. Furthermore, stabilizers of dodecyltrimethylammonium bromide (DTAB) and didodecyldimethylammonium bromide (DDAB) were used to make polyhexylmethacrylate NPs with a size range of 38 to 53 nm [297]. SABS-8 and SABS-10, two polymerizable anionic surfactants, were employed successfully in microemulsion polymerization of butyl methacrylate (BMA) at room temperature utilizing the redox initiator ammonium persulfate (APS)/tetramethylethylenediamine (TMEDA) [298]. Some other work used the cationic surfactant decyltrimethylammonium bromide DeTAB to make polypyrrole NPs with a particle size of 2 nm [299]. The polymerization of butyl acrylate with a sodium dodecyl sulfate/Aerosol OT surfactant combination and potassium peroxodisulfate as an initiator yielded particles smaller than 40 nm [300].

## 3. Biologically Synthesized Biodegradable Polyhydroxyalkanoate-Based Nanoparticles

A non-toxic, reliable, and eco-friendly experimental protocol for the synthesis of NPs is highly in demand. Natural entities such as secondary metabolites, enzymes, polysaccharides, biodegradable polymers, vitamins, and microorganisms can be utilized for the synthesis of NPs [95,301]. One such promising approach is the biosynthesis of NPs using bacteria. To date, a large variety of bacterial species have already been studied in the hopes of developing alternate NP synthesis techniques. For the time being, scientists are producing NPs using bacteria’s biomass or cell extracts [302]. In comparison to other biological entities, bacteria are thought to be a promising biofactory for the synthesis of NPs. Bacterial biosynthesis of NPs is a fast-growing study area in the field of science and nanotechnology, with many species of bacteria being used to synthesize NPs all around the world [303].

**Figure 8:**
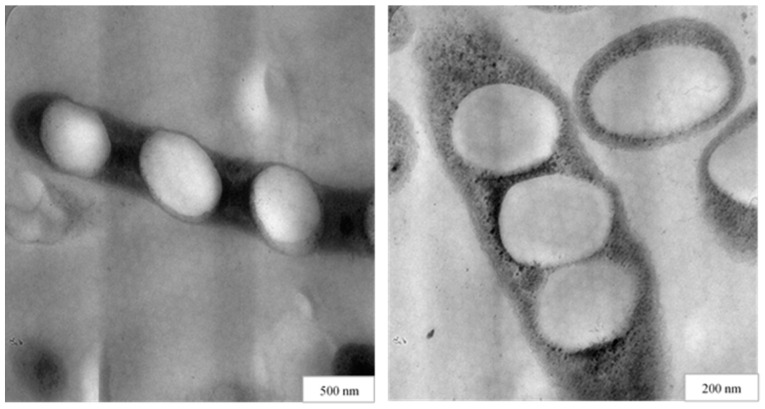
TEM image of nanosized PHA granules inside bacterial cells.

PHA is one of the PNPs generated spontaneously in the bacterial cytoplasm. PHA belongs to the aliphatic polyesters family of biodegradable and biocompatible polymers [304,305,306]. PHA is produced spontaneously by some bacteria in the form of nanosized granules under unbalanced growth conditions, such as an excess of carbon source and nutritional limitations, such as nitrogen, oxygen, and phosphorus [307,308,309]. Figure 8 depicts TEM images of nanosized PHA granules inside bacterial cells. Numerous parameters can affect the size of PHA granules such as PHA granule-associated proteins (phasins), bacterial species or strains, cultivation conditions, and time [310,311,312,313,314]. PHA granules made this way are tunable (for chemical composition, size, or other key qualities) to levels not possible with chemical synthesis, genetically altering the bacterial strain or adjusting the production circumstances such as the culture media composition. The crux of interest that lies in PHA NPs is their self-assembling properties, their production using easily cultivated bacterial species, and the different morphological types of particles [315].

P(3HB-*co*-3HV) nanocarriers were used by Williams et al. for the controlled release of tetracycline [316]. In the hepatocellular carcinoma cell BEL7402, the PHA granules binding protein PhaP has been used in a receptor-mediated drug carrier using RBITC as a drug delivery model created using the modified emulsification/solvent diffusion approach [317]. Meanwhile, P(3HB) NPs with 55 nm average diameter were used for encapsulating retinoic acid through the dialysis method [318]. Shishatskaya et al. studied P(3HB) incorporated with rubomycin in vivo, and it was found that rubomycin-loaded PHB fabricated using the solvent evaporation method was effective in arresting carcinoma proliferation and thus, increased mice survival [319]. P(3HB-*co*-3HO) was employed as the drug carrier in targeted drug delivery. In another study, a new nanocarrier was formulated with folic acid (FA) and doxorubicin (DOX) as the targeting ligand and anticancer drug, respectively. This nanocarrier was found to be a potential candidate for the targeted delivery of anticancer drugs to the folate receptor-overexpressed cancer cells [320]. Sulperazone-loaded P(3HB-*co*-3HV) was employed for an in vitro antibiotic release [321]. Rossi et al. studied the release profile of gentamycin incorporated into P(3HB-*co*-3HV) and found that the copolymer with higher HV content released more gentamycin [322]. Additionally, P(3HHx-*co*-3HO) NPs were found to facilitate the permeation of tamsulosin drugs into the skin [323].

## 4. Conclusions and Future Perspectives

Different methods for the formation of PNPs were discussed in this review, including the dispersion of preformed polymers (emulsification solvent evaporation, nanoprecipitation, emulsification solvent diffusion, and salting-out) and the polymerization of monomers (emulsion polymerization, surfactant-free emulsion polymerization, mini-emulsion polymerization, and micro-emulsion polymerization). Furthermore, the effects of experimental variables on the characteristics of the formed PNPs were discussed. PNPs have a wide variety of applications; however, there are many challenges associated with the synthesis of PNPs that need to be addressed before PNPs can be fully utilized and integrated into applications, for example, the ability to reproduce PNPs with a good size distribution. The size distribution of PNPs obtained using currently existing methods is usually very broad and is not suited for most applications. In addition, there are not many reports on the scaled-up production of PNPs. Many scientific reports have also claimed that the synthesis of PNPs at a lab scale exists only as a proof of concept of the technology. Many produced and evaluated NPs never reach clinical trials due to their non-biocompatible physiochemical properties. For this reason, PHA NPs are ideal for use as drug carriers as they abide by the present regulatory requirements in terms of biodegradability, stability, and non-toxicity. Furthermore, because the size distribution is extremely large, the particle size loses certainty due to the wide range of size distribution. This situation poses a great challenge in using PNPs for drug delivery applications. Furthermore, PHA NPs can be produced using bacteria, which allows for green synthesis to produce nanocarriers that can be used extensively in the field of nanomedicine. With regards to application, PNPs can be used as drug carriers to target specific sites within cells or organs for more advanced treatment due to their unique properties and size. This would greatly improve the performance of targeted therapies. The PNPs can also be used for diagnostic purposes, either in the lab or in hospitals (point-of-care diagnostics).

## Figures and Tables

**Table 1:** Advantages and limitations of two types of polymer synthesis methods; dispersion of preformed polymer and direct polymerization.

Method	Advantages	Limitations	References
Dispersion of preformed polymers			
nanoprecipitation	Requires low energy Reproducible Single step Scalability	Size of NPs can be affected by stirring rate Low efficiency of drug encapsulation	[78,79]
emulsification solvent evaporation	Scalability Single step emulsion for hydrophobic agents Double or multiple step emulsion for hydrophilic agents	Requires heating or vacuum for evaporation Residual solvent or stabilizer Not stable	[80,81]
emulsification solvent diffusion	Does not require homogenizer High reproducibility Easy to scale up	Uses high volumes of water Probable leakage of water-soluble drugs into external phase Lower efficiency in lipophilic drug encapsulation	[82,83]
salting out	Does not require heating Avoids chlorinated solvents Suitable for DNA, RNA, and proteins	Requires high speed homogenization Exclusive for the encapsulation of lipophilic drugs Time-consuming Limited scalability	[84,85]
Direct polymerization			
emulsion	Produce polymers with high molar mass Uses water as dispersion medium Excellent heat dissipation	Requires removal of surfactant Time consuming High cost	[86,87]
surfactant-free emulsion	Does not require surfactant Simple and green process Uses water-soluble initiators	Requires the preparation of monodisperse and uniformly distributed particle sizes	[88,89]
mini emulsion	Uses a low molecular mass co-stabilizer Small particle size Low volume of surfactant	Uses a high-shear device Surfactant is retained in the polymer	[90,91]
micro-emulsion	Uses water-soluble initiators Thermodynamically stable	Formation of empty micelles Destabilized microdroplets Increased particle size Requires a high ratio of surfactant	[92,93]
microbial	Non-toxic Eco-friendly Biocompatible	High production cost	[94,95]

## Data Availability

Data sharing is not applicable for this review.
